# Is there a mental health diagnostic crisis in primary care? Current research practices in global mental health cannot answer that question

**DOI:** 10.1017/S2045796025000010

**Published:** 2025-01-30

**Authors:** Brandon A. Kohrt, Dristy Gurung, Ritika Singh, Sauharda Rai, Mani Neupane, Elizabeth L. Turner, Alyssa Platt, Shifeng Sun, Kamal Gautam, Nagendra P. Luitel, Mark J.D. Jordans

**Affiliations:** 1 Center for Global Mental Health Equity, Department of Psychiatry and Behavioral Health, George Washington University, Washington, DC, USA; 2 Research Department, Transcultural Psychosocial Organization Nepal (TPO Nepal), Kathmandu, Nepal; 3 Department of Biostatistics and Bioinformatics and Duke Global Health Institute, Duke University, Durham NC, USA; 4 Health Service and Population Research Department, Institute of Psychiatry, Psychology and Neuroscience, Center for Global Mental Health, King’s College London, London, UK

**Keywords:** depression, developing countries, diagnosis, global health, primary care, psychiatric status rating scales, psychosis, screening

## Abstract

In low- and middle-income countries, fewer than 1 in 10 people with mental health conditions are estimated to be accurately diagnosed in primary care. This is despite more than 90 countries providing mental health training for primary healthcare workers in the past two decades. The lack of accurate diagnoses is a major bottleneck to reducing the global mental health treatment gap. In this commentary, we argue that current research practices are insufficient to generate the evidence needed to improve diagnostic accuracy. Research studies commonly determine accurate diagnosis by relying on self-report tools such as the Patient Health Questionnaire-9. This is problematic because self-report tools often overestimate prevalence, primarily due to their high rates of false positives. Moreover, nearly all studies on detection focus solely on depression, not taking into account the spectrum of conditions on which primary healthcare workers are being trained. Single condition self-report tools fail to discriminate among different types of mental health conditions, leading to a heterogeneous group of conditions masked under a single scale. As an alternative path forward, we propose improving research on diagnostic accuracy to better evaluate the reach of mental health service delivery in primary care. We recommend evaluating multiple conditions, statistically adjusting prevalence estimates generated from self-report tools, and consistently using structured clinical interviews as a gold standard. We propose clinically meaningful detection as ‘good-enough’ diagnoses incorporating multiple conditions accounting for context, health system and types of interventions available. Clinically meaningful identification can be operationalized differently across settings based on what level of diagnostic specificity is needed to select from available treatments. Rethinking research strategies to evaluate accuracy of diagnosis is vital to improve training, supervision and delivery of mental health services around the world.

## Introduction

Integration of mental health services in primary care has been identified as a key strategy to reduce the global mental health treatment gap (Patel *et al.*, 2018; World Health Organization, 2022). The World Health Organization (WHO) *Comprehensive Mental Health Action Plan* calls for 80% of countries to have integration of mental health services in primary care by 2030 (World Health Organization, 2021). Currently, in most low- and middle-income countries (LMIC), primary healthcare workers, including physicians, nurses and auxiliary staff, receive either no exposure or only minimal exposure to mental healthcare in their pre-service training (World Health Organization, 2020). To address this gap, brief in-service educational programmes, such as the five-day curriculum for WHO’s mental health Gap Action Programme-Intervention Guide (mhGAP-IG), have been implemented in over 90 countries to facilitate the integration of mental health services into primary care (Brohan *et al.*, 2024; Keynejad *et al.*, 2021; World Health Organization, 2016).

A shortcoming of these in-service training programmes has been the lack of accurate identification of patients who need mental health services. Fewer than 1 in 10 people with depression are diagnosed by primary healthcare workers, based on a recent systematic review (Fekadu *et al.*, 2022), and services are similarly limited for other conditions (Alonso *et al.*, 2018; Degenhardt *et al.*, 2017; Jenkins *et al.*, 2013; Kauye *et al.*, 2014). For primary care-based programmes to be successful, healthcare workers in these settings need to improve accurate detection of mental health conditions.

Unfortunately, the current research methods of assessing diagnostic accuracy are inadequate and potentially misleading. In this commentary, we describe the current strategies for evaluating diagnostic accuracy. We draw attention to weaknesses, notably reliance on self-report tools and a focus on depression rather than working across mental health conditions. We propose an alternative research approach focusing on multiple conditions using more accurate statistical estimation of prevalence from self-report tools combined with greater integration of structured clinical interviews. We discuss how classification of accurate diagnoses needs to be context specific, arguing that research using ‘good-enough’ diagnoses will inform training, supervision and implementation of mental health interventions to improve reach of services and minimize risk of harm from incorrect diagnoses.

## Limitations of current approaches to estimating rates of accurate diagnoses

### Limitation 1: False positive rates of self-report tools

Self-report screening tools are commonly used as the reference standard when determining whether or not a primary healthcare worker has accurately diagnosed a mental health condition (Fekadu *et al.*, 2022; Habtamu *et al.*, 2023; Rathod *et al.*, 2016). For example, when judging if a primary healthcare worker accurately diagnosed depression, the score on the Patient Health Questionnaire-9 (PHQ-9; Kroenke *et al.*, 2001) has become a *de facto* standard (Fekadu *et al.*, 2022; Habtamu *et al.*, 2023). The percentage detection rate is calculated as the number of patients who receive a diagnosis of depression by a healthcare worker compared to the number of patients above a locally validated cut-off on the self-report screening tool. A patient with a high PHQ-9 score who does not receive a depression diagnosis by a primary healthcare worker is considered a missed diagnosis.

This strategy is problematic because self-report tools are not synonymous with a clinical diagnosis. Instead, the gold standard for clinical diagnosis is a semi-structured clinician-administered interview, using tools such as the Structured Clinical Interview for the Diagnostic and Statistical Manual of Mental Disorders (SCID; First *et al.*, 2015) or the Scheduled for Affective Disorders and Schizophrenia for School Aged Children (Kiddie-SADS; Kaufman *et al.*, 2016). When self-report tools are compared against these structured clinical interviews, the self-report tools typically have high rates of false positives: they identify many people who do not have the clinical condition, i.e., low specificity (Levis *et al.*, 2020). This is by design because most self-report tools were created to improve screening and referral in health services, and they were not intended to provide a diagnosis (Zimmerman and Holst, 2018). Administration of self-report tools typically prioritizes sensitivity – capturing the greatest number of individuals who potentially have a condition, even if that has the tradeoff of high rates of false positives.

In the recent review of depression detection rates in LMIC, most studies used a PHQ-9 cut-off of 5 or 10 to estimate who should have received a clinical diagnosis of depression (Fekadu *et al.*, 2022). The DEPRESS-D research consortium has conducted large individual participant meta-analyses of the PHQ-9 versus structured clinical interviews (Levis *et al.*, 2020). They demonstrated that the commonly used cut-off of ≥ 10, results in two-fold inflation of the actual prevalence (12% prevalence based on the SCID compared to 24% on the PHQ-9 ≥ 10): half of the patients above the cut-off do not have clinical condition when evaluated with structured clinical interviews (Levis *et al.*, 2020). Therefore, using self-report tools creates a misleading target – often an overestimate – of the number of expected diagnoses (Aragonès *et al.*, 2006; Zimmerman and Holst, 2018). The DEPRESS-D group summarizes this problem:

Reporting this percentage [above the PHQ-9 cut-off] as depression prevalence, however, would be akin, for example, to reporting the proportion of women with positive mammogram screens as the prevalence of breast cancer and… would dramatically overestimate prevalence. (Levis *et al.*, 2020)

In the context of evaluating diagnostic accuracy, this translates into the PHQ-9 and similar tools overestimating the number of expected diagnoses in primary care. This incorrectly inflates the true difference between the rate of healthcare diagnoses and the target number of diagnoses to be made. In other words, it can make the gap in detection by healthcare workers appear worse than it actually is.

### Limitation 2: False negative rates of self-report tools

Self-report tools are also not 100% sensitive. Some patients with clinical depression will score below cut-offs – a false negative. A competent primary healthcare worker would be expected to make some diagnoses of depression below the cut-off and to not diagnose every patient above the cut-off. When only examining diagnoses of depression among patients scoring above a PHQ-9 cut-off, this misses those clinical cases with depression scoring below the cut-off. This group of screener-negative depression cases is lost in both the numerator and denominator of percent detection. The PHQ-9 and other self-report tools used in isolation are, therefore, unable to provide a true estimate of percent detection by healthcare workers.

### Limitation 3: Use of tools that are not validated for local populations

A recent review of diagnostic error in mental health points out that “validated psychological tests … can lead to inaccurate diagnostic impressions if they are interpreted without sufficient context or not followed with an appropriate diagnostic interview” (Bradford *et al.*, 2024). This leads to another problem with the predominance of self-report tools: the issue of local validation. In global mental health, self-report tools require translation and appropriate cultural adaptation, followed by validation to establish the local estimates for sensitivity and specificity (Kohrt and Kaiser, 2021; Kohrt and Patel, 2020; Van Ommeren, 2003; Van Ommeren *et al.*, 1999). Without local validation, the rates of false positives and false negatives of the self-report tool cannot be accurately determined. This further exacerbates error in estimating targets for clinician diagnoses.

### Limitation 4: Focusing on a single mental health condition

**Figure 1.**
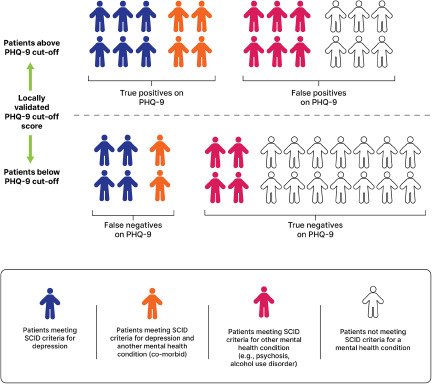
Heterogeneity of the patient population under the categorization of above versus below cut-off on a self-report mental health screening tool in comparison to a gold standard diagnosis using the SCID. Abbreviations: PHQ-9, patient health questionnaire-9; SCID, structured clinical interview for the diagnostic and statistical manual of mental disorders.

Another limitation is that studies of diagnostic accuracy rarely evaluate multiple mental health conditions. Using a tool such as the PHQ-9 does not allow for distinguishing among conditions that may be misdiagnosed as depression. PHQ-9 scores are likely to be high among patients with generalized anxiety, posttraumatic stress, a substance use condition, or negative symptoms of psychosis. Physical health conditions including anaemia, other nutrient deficiencies, hypothyroidism, and infectious diseases may also have high PHQ-9 scores (Bode *et al.*, 2021; Califf *et al.*, 2022). The PHQ-9 basically functions like a thermometer suggesting that a fever is present, but the tool used in isolation cannot distinguish which condition is causing the fever. Conflating every high PHQ-9 score with a clinical diagnosis of depression is like assuming every fever is malaria. Consequently, evaluating healthcare workers’ ability to identify depression requires clinical assessment of multiple mental health conditions. Figure 1 illustrates the high number of false positives using the PHQ-9 and heterogeneity underlying a categorical classification depression based on a commonly used PHQ-9 cut-off score.

There are self-report tools with multiple conditions, such as the Diagnostic and Statistical Manual of Mental Disorders (DSM)-5 Level 1 Cross-Cutting Symptom Measure (DSM-XC), which addresses 13 mental health domains (American Psychiatric Association, 2013). However, this tool has not been validated in most settings. In data from Brazil, the domain subscales suffer from many of the problems of single condition tools, for example even lower specificity than the PHQ-9 (DSM-XC specificity: major depressive disorder = 59%, generalized anxiety disorder = 54%, alcohol use disorder = 55%), leading to high rates of false positives (Gonçalves Pacheco *et al.*, 2024). The domains are also sensitive across multiple conditions, e.g., the depression domain has a sensitivity of 95% for major depressive disorder and 80% for generalized anxiety disorder (Gonçalves Pacheco *et al.*, 2024). Considering these findings, the DSM-XC is unable to meet the objective of distinguishing among conditions as a benchmark for diagnostic accuracy.

## Strategies for improving research to evaluate diagnostic accuracy

### Strategy 1. Statistical techniques to adjust estimates from self-report tools

Self-report tools have the advantage of being brief and not requiring clinical experts for administration. However, adjustments are required to address the limitations described above. Self-report tools need to be validated in the population of interest using structured clinical interviews to determine the psychometric properties (Kohrt and Kaiser, 2021; Kohrt and Patel, 2020). Based on the validation, sensitivity and specificity can also be evaluated at different cut-offs with the target population. The DEPRESS-D group reports that selecting PHQ-9 cut-offs higher than 10 can be associated with more accurate prevalence rates by minimizing false positives (Levis *et al.*, 2020, 2019). Tools such as the PHQ-9 also have diagnostic algorithms to estimate DSM diagnoses (Levis *et al.*, 2020). In a sample of 1,900 primary care patients in Nepal, a PHQ-9 cut-off of ≥10 yielded a prevalence rate of 14.5% compared to 5.6% when using the DSM algorithm for PHQ-9 scoring (Luitel *et al.*, 2024a). Although overall prevalence rates may be closer to the true population prevalence when using scoring algorithms for DSM equivalence, the classification accuracy of DSM algorithm scoring does not appear to be better than the PHQ-9 sum scores (He *et al.*, 2019; Levis *et al.*, 2020).

After a scoring strategy and cut-off are selected, the sensitivity and specificity can be used to calculate the ‘true prevalence rate’ (TPR). This is done by estimating the number of false positives above the cut-off and false negatives below the cut-off, then adjusting the prevalence. This approach is well known in epidemiology (Hennekens *et al.*, 1987), and it has been used in infectious disease research to generate more accurate estimates (Bentley *et al.*, 2012). However, it has rarely been used with mental health data (Carvajal-Velez *et al.*, 2023; Luitel *et al.*, 2024b; Marlow *et al.*, 2023; Tele *et al.*, 2023). Unfortunately, this approach does not work when disease prevalence is low and the tool has a low specificity. In these instances, the number of expected false positives can lead to estimated TPR that is negative. Therefore, newer strategies using Bayesian statistics can provide more accurate estimates in the setting of low prevalence (Diggle, 2011), and some strategies can be used when sensitivity and specificity are not known for the local setting (Lewis and Torgerson, 2012). It is important to note that all of these statistical adjustments will contribute to a more accurate estimated target rate for the overall prevalence in a primary care population, but, without further clinical information, it does not improve the diagnostic categorization of an individual patient.

### Strategy 2. Integrating structured clinical interviews

Self-report tools can be a useful starting point to evaluate detection, but additional methods are needed to make judgements of accurate diagnosis. Structured clinical interviews are semi-structured guides utilized by mental health clinicians such as psychiatrists and clinical psychologists. The SCID (First *et al.*, 2015) and K-SADS (Kaufman *et al.*, 2016) are commonly used in clinical research to ensure inclusion and exclusion criteria for a new medication or other treatment. They can be used to determine accuracy of diagnosis for specific patients. These tools have branching logic that enable assessment across the diagnostic spectrum, as well as identification of co-occurring conditions, i.e., psychiatric comorbidity. Structured clinical interviews include sections to evaluate when conditions are likely secondary to substance use or another medical condition. Mental health experts using structured clinical interviews can also use their own clinical judgement when the algorithms may not capture nuanced clinical presentation, as well as adjust diagnostic judgements based on cultural context as it relates to clinical relevance of symptoms and functioning (Sajida Abdul and Panos, 2008). Structured clinical interviews are time intensive. Clinicians also need training on using the guides, including establishing inter-rater reliability because of the subjectivity and semi-structured nature of the guide (De La Peña *et al.*, 2018; Kolaitis *et al.*, 2003).

Given the resources required for structured clinical interviews, a feasible approach may be to use a two-stage strategy in which self-report tools are used for a large study sample and structured clinical interviews are conducted with select subsamples after collection of self-report data (Kauye *et al.*, 2014). This approach has been recommended in other fields of medicine, especially when evaluating populations with a low prevalence of the target health conditions (Obuchowski and Zhou, 2002). In this approach, in the first stage, self-report tools could be administered to a large representative sample of primary care patients. Then in the second stage, a smaller subsample selected for structured clinical interviews would include a mix of individuals who received mental health diagnoses from primary healthcare workers and those who did not receive a diagnosis but who scored above validated cut-offs on the self-report tools administered in the first tier. This would generate diagnostic accuracy estimates mitigating the high rates of false positives in self-report measures. The structured clinical interview administered to a subsample of individuals who did not receive a diagnosis from a healthcare worker and were below the cut-off could reduce the estimated number of false negatives. The subsampling weights could then be used to estimate the prevalence rate in the full original population that completed only the self-report tools.

### Strategy 3. Classifying ‘good-enough’ diagnostic accuracy based on contexts of services

Integrating structured clinical interviews with self-report tools adds complexity for classifying what counts as diagnostic accuracy. It is neither realistic nor clinically necessary that primary healthcare workers diagnose patients exactly as they would be categorized by a structured clinical interview. For example, it is unreasonable to expect that a primary healthcare worker after one week of mental health training should achieve SCID-level distinctions among major depressive disorder, cyclothymia and adjustment disorder with depressed mood. Therefore, rather than focusing on perfect diagnostic matches, we propose a flexible approach with ‘good-enough’ diagnostic synergy between a primary healthcare worker’s conclusion and structured clinical interview outcomes. Good-enough diagnoses will vary based on the types of treatments available, the potential risks associated with different conditions and treatments, and the social implications of misdiagnosis. Good-enough does not refer to allowing for a certain percentage of errors, but instead it reflects that diagnoses from a class of similar conditions may be close-enough to count as correct because the treatments are similar.

In LMICs, the range of available mental health treatments is limited. Pharmacological and psychological interventions recommended for depression and anxiety overlap, suggesting that a primary healthcare worker’s diagnosis of one condition could be adequate even if the clinical diagnosis is the other (Patel, 2001). Conversely, for conditions with higher-risk treatment implications, such as psychosis, diagnostic specificity becomes important. A misdiagnosis of psychosis may lead to the prescription of antipsychotic medications, which carry significant potential for adverse effects for persons who do not have the condition (Coulter *et al.*, 2019). This has heightened importance in resource-limited settings, where patients often lack regular access to follow-up care to monitor and mitigate potentially incorrect treatments.

**Figure 2.**
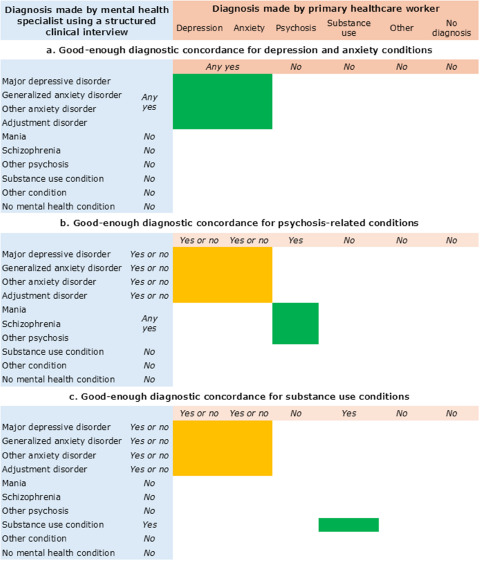
Examples of ‘good-enough’ diagnostic concordance between mental health specialist’s structured clinical interview and primary healthcare worker’s diagnosis. Green sections refer to required concordance, and yellow sections can be discordant. (a) Depression or anxiety conditions can be considered accurate with any combination of depression or anxiety diagnoses because of the similar treatment in low-resource settings. (b) Psychosis diagnoses by healthcare workers would be accurate if any of the psychosis related conditions are positive on the structured clinical interview, including mania, schizophrenia or other psychosis, regardless of any discordance on the depression and anxiety conditions. (c) Substance use conditions require concordance with the structured clinical interview, but discordance on depression and anxiety conditions is acceptable.

**Figure 3.**
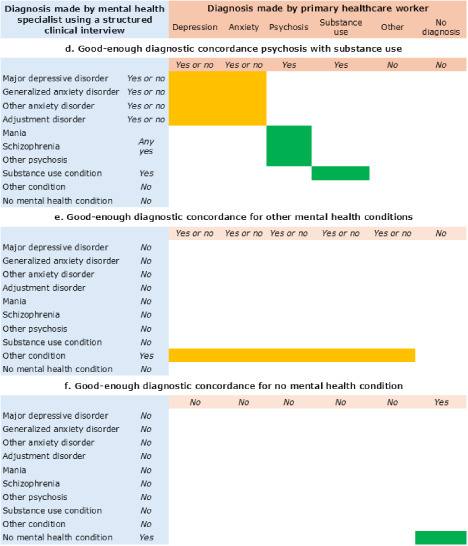
Additional examples of ‘good-enough’ diagnostic concordance: (d) for substance use conditions co-occurring with psychosis, this requires that both the substance use condition and psychosis would be indicated, e.g., alcohol withdrawal with features of psychosis, acute intoxication with a substance with psychotic features, or persons with psychosis who have a comorbid substance use condition. (e) For other conditions, this will depend on the condition and context regarding what is considered an acceptable overlap, e.g., PTSD on the structured clinical interview could be acceptable if depression or anxiety is diagnosed by the healthcare worker because of similar treatment. (f) For no mental health condition, there must be agreement between the clinician’s interview and healthcare worker’s diagnosis that no mental health treatment is needed.

The WHO mhGAP-IG is an example of simplifying diagnostic categories for a good-enough approach to clinical care (World Health Organization, 2016). The mhGAP-IG uses streamlined diagnostic categories that allow primary healthcare workers to treat mental health conditions without necessitating exhaustive distinctions. The diagnostic categories in mhGAP-IG 2.0 are depression, psychosis, epilepsy, dementia, disorders due to substance use, self-harm/suicide, other significant mental health complaints and child and adolescent mental and behavioural disorders (World Health Organization, 2016). The psychosis module includes both psychosis and mania, and they may be treated similarly with antipsychotics when other options are not available. Similarly, in the first two versions of mhGAP-IG, there was not a separate module for anxiety. For many anxiety conditions, treatment is comparable to depression guidelines for psychotherapy and/or SSRIs. In summary, diagnostic distinctions can be adjusted based on the treatments available. **Figures 2** and **3** provide an example of categorizing good-enough diagnoses when working with categories of depression, anxiety, psychosis and alcohol use disorder in a low-resource setting.

To guide good-enough diagnostic accuracy research, we propose four considerations for what may constitute clinically meaningful diagnoses within primary care settings. First, determine whether specific treatment outcomes are contingent on an exact diagnosis, especially when available treatments overlap across diagnostic categories. Diagnoses should parallel the specificity needed for treatment within each setting, recognizing that a few simplified diagnostic categories may suffice if resources are constrained. Second, assess the risk associated with treatment, as higher-risk treatments warrant stricter diagnostic precision. Third, consider the social implications of diagnoses, as misdiagnoses that lead to social harm demand more careful evaluation. Finally, evaluate the resource implications of both incorrect diagnoses (false positives) and missed diagnoses (false negatives) to balance diagnostic thoroughness with sustainable use of healthcare resources.

## Conclusion

To improve diagnostic accuracy, global mental health research must move beyond relying solely on self-report screening tools as the benchmark for a clinical condition. Combining statistical adjustment of self-report tool prevalence rates with structured clinical interviews offers a more robust approach, enabling us to assess how well primary healthcare workers are performing and to enhance their training, supervision and programme implementation. Accurate diagnosis is critical not only to identify those in need but also to avoid the potential harm of unnecessary or inappropriate treatments. In global mental health, achieving clinically meaningful diagnostic accuracy also requires a shift away from strict adherence to the full suite of psychiatric categories and instead should move towards culturally and contextually relevant good-enough diagnostic categorization. This flexibility empowers primary healthcare workers to deliver effective, safe and socially responsible care, ultimately bridging the global mental health treatment gap.
